# Intrinsic calcification angle: a novel feature of the vulnerable coronary plaque in patients with type 2 diabetes: an optical coherence tomography study

**DOI:** 10.1186/s12933-019-0926-x

**Published:** 2019-09-24

**Authors:** Sebastian Reith, Andrea Milzi, Enrico Domenico Lemma, Rosalia Dettori, Kathrin Burgmaier, Nikolaus Marx, Mathias Burgmaier

**Affiliations:** 1 Department of Cardiology, Medical Clinic I, University Hospital of the RWTH Aachen, Pauwelsstr. 30, 52074 Aachen, Germany; 2 Zoological Institute, Department of Cell- and Neurobiology, Karlsruhe Institute of Technology (KIT), Karlsruhe, Germany; 3 Department of Pediatrics, University Hospital of Cologne, Cologne, Germany

**Keywords:** Coronary calcification, Plaque vulnerability, Optical coherence tomography, Coronary artery disease, Atherosclerosis, Diabetes mellitus

## Abstract

**Background:**

Coronary calcification is associated with high risk for cardiovascular events. However, its impact on plaque vulnerability is incompletely understood. In the present study we defined the intrinsic calcification angle (ICA) as the angle externally projected by a vascular calcification and analyzed its role as novel feature of coronary plaque vulnerability in patients with type 2 diabetes.

**Methods:**

Optical coherence tomography was used to determine ICA in 219 calcifications from 56 patients with stable coronary artery disease (CAD) and 143 calcifications from 36 patients with acute coronary syndrome (ACS). We then used finite elements analysis to gain mechanistic insight into the effects of ICA.

**Results:**

Minimal (139.8 ± 32.8° vs. 165.6 ± 21.6°, p < 0.001) and mean ICA (164.1 ± 14.3° vs. 176.0 ± 8.4°, p < 0.001) were lower in ACS vs. stable CAD patients. Mean ICA predicted ACS with very good diagnostic efficiency (AUC = 0.840, 95% CI 0.797–0.882, p < 0.001, optimal cut-off 175.9°); younger age (OR 0.95 per year, 95% CI 0.92–0.98, p = 0.002), male sex (OR 2.18, 95% CI 1.41–3.38, p < 0.001), lower HDL-cholesterol (OR 0.82 per 10 mg/dl, 95% CI 0.68–0.98, p = 0.029) and ACS (OR 14.71, 95% CI 8.47–25.64, p < 0.001) were determinants of ICA < 175.9°. A lower ICA predicted ACS (OR for 10°-variation 0.25, 95% CI 0.13–0.52, p < 0.001) independently from fibrous cap thickness, presence of macrophages or extension of lipid core. In finite elements analysis we confirmed that lower ICA causes increased stress on a lesion’s fibrous cap; this effect was potentiated in more superficial calcifications and adds to the destabilizing role of smaller calcifications.

**Conclusion:**

Our clinical and mechanistic data for the first time identify ICA as a novel feature of coronary plaque vulnerability.

## Background

Coronary artery calcification (CAC) is a known feature of atherosclerosis [1] and extensive CAC is associated with a higher risk for cardiovascular events [1–3]. However, it has recently been suggested that not only CAC extent, but also its morphology may play a causal role in the development of acute coronary syndromes (ACS) [4, 5]. In fact, studies assessing the mechanics of the coronary plaque in the presence of microcalcifications highlighted that these small inclusions cause a significant increase in the peak circumferential stress exerted on the fibrous cap [6–8]. Such characteristics are only partially detected by computed tomography, which is being increasingly used in cardiovascular prevention in order to assess CAC extent; on the other hand, optical coherence tomography (OCT) is used in studies assessing CAC morphology because of its excellent resolution and ability to detect calcifications [9–13]. Using intravascular imaging, previous studies found spotty calcifications, i.e. calcifications with a calcium arc < 90°, to be more frequent in patients with ACS compared to patients with stable coronary artery disease (stable CAD) [14–17] and to predict future revascularization [18]. These data, together with the previously cited mechanistic analyses [6–8], led to the hypothesis that small calcifications yield a destabilizing effect on coronary plaques, as opposed to the stabilizing effects of larger calcifications. However, this view is not unchallenged, as there are studies which—albeit in small populations—could not determine any difference in CAC morphology between ACS and stable CAD patients [10].

However, very little is known about morphological parameters of CAC besides the size of calcification as potential contributing factors to plaque vulnerability. Cardoso et al. employed finite elements analysis on ex vivo samples and could demonstrate that a prolate spheroid morphology of microcalcifications further increases the stress on the fibrous cap [19]. Still, nothing is known about the geometrical properties of CAC which may influence plaque vulnerability. Thus, we aimed to analyze this aspect from a both clinical and mechanistic viewpoint using OCT and finite elements structural analysis.

To the best of our knowledge, we are first—among both theoretical models and in vivo studies—to 1. define the intrinsic calcification angle (ICA, i.e. the angle externally projected by a vascular calcification) and to 2. test it as a novel parameter of plaque vulnerability in patients with type 2 diabetes. We selected a diabetic cohort because of its high cardiovascular risk and the high prevalence of vulnerable plaques in these patients.

## Methods

### Study population

In this study we analyzed 362 coronary calcifications from the target/culprit segments of 92 patients with type 2 diabetes mellitus (T2DM) who underwent coronary angiography in the Department of Cardiology at the University Hospital of the RWTH Aachen. Inclusion criteria were presence of an at least intermediate coronary stenosis suitable for OCT—analysis, presence of CAC in the target/culprit segment as well as T2DM diagnosis. Definition of T2DM was based on an HbA1c > 6.5% and/or current antidiabetic therapy [20]. Detail of screening and inclusion are reported in Additional file 1: Figure S1. A subgroup of this population was part of previously published analyses [12, 13].

Among the total 362 calcifications, 219 were from 56 patients with stable CAD and 143 from 36 patients with ACS. Stable CAD was defined as no progression of frequency and duration of clinical symptoms within the 6 weeks preceding coronary angiography. In this case, the coronary target lesion was identified through echocardiographic wall motion abnormalities, positive stress testing by MRT or echocardiography, and/or pathological fractional flow reserve (≤ 0.80) measurements. The ACS population was composed of patients with NSTEMI, defined as acute chest pain without ST-segment elevation and increased high-sensitivity cardiac troponin. Patients presenting with suspected type 2 myocardial infarction were not included in the present study.

Exclusion criteria were the localization of the lesion in the left main coronary artery, at a vessel bifurcation, in a previously implanted stent or in a bypass graft, as well as ongoing cardiogenic shock, acute or chronic kidney disease (with serum creatinine > 1.5 mg/dl) or pregnancy. Glomerular filtration rate was estimated using the MDRD formula. Pre-procedural written informed consent of all patients was obtained. The study was approved by the local ethics committee (EK 071/11 and EK 277/12) and is in accordance with the declaration of Helsinki on ethical principles for medical research involving human subjects.

### Image acquisition

The OCT images were acquired prior to coronary intervention in the coronary target/culprit segment using a frequency domain OCT C7XR system and the DragonFly catheter (St. Jude Medical Systems; Lightlab Imaging Inc., Westford, Massachusetts, USA). If necessary, pre-dilatation was performed in order to allow advancement of the OCT catheter. Pre-dilatation was performed on the interventionalist’s discretion and had an overall prevalence < 10%. Thrombus aspiration or thrombolysis were not performed in any patient. We obtained blood removal by the injection of 14 ml contrast dye (iodixanol) at a flow rate of 4 ml/s through the guiding catheter. Image acquisition was obtained with automated pull-back rate of 20 mm/s. Plaque analysis was performed offline by two independent observers throughout the target/culprit segment, defined as the whole length of the OCT pullback containing the target/culprit lesion stenosis, frame by frame in 0.2 mm intervals utilizing St. Jude’s proprietary software.

### OCT-based morphological analysis of calcification

In accordance to the standards for plaque morphology assessment using OCT, calcifications were defined as signal-poor heterogenous regions with clearly delineated contours [21]. Two calcifications were considered distinct when no continuity between them could be detected, i.e. when they were located on different portions of the vessel wall without any contact throughout their length or when they were longitudinally separated for at least 1 mm.

**Fig. 1:**
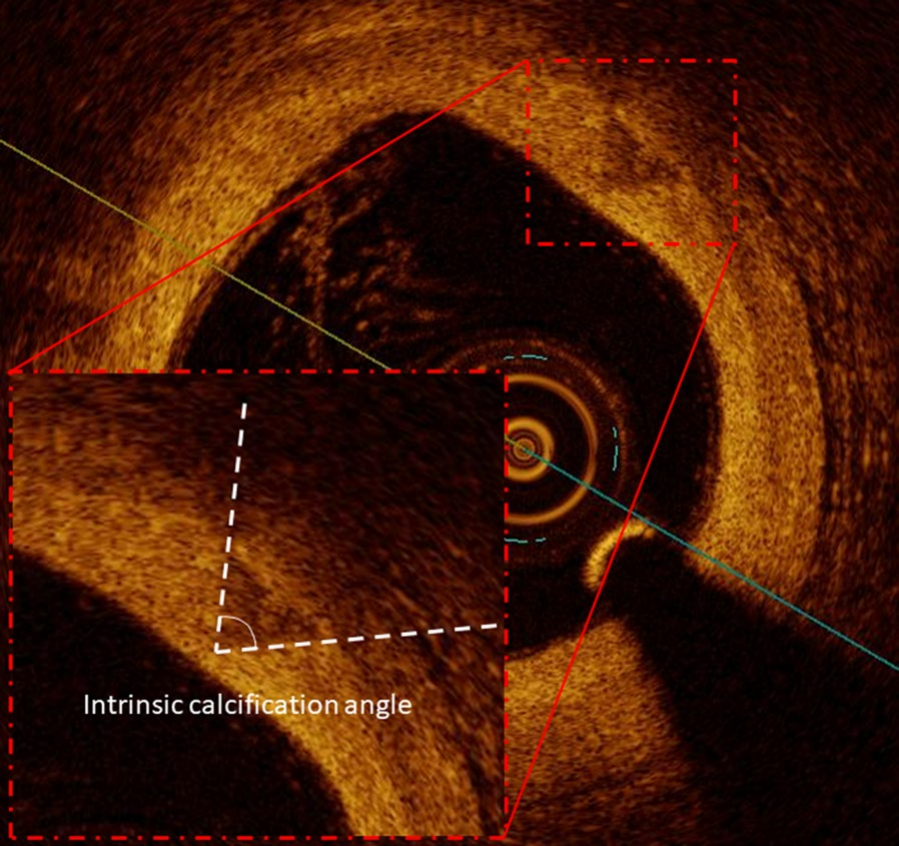
OCT–based measurement of ICA. The calcification is marked with a red square and is magnified. In this image, the angle abluminally projected by the calcification, i.e. the ICA, is marked. *ICA* intrinsic calcification angle

Morphologic parameters of CAC were assessed as previously described [12]. Furthermore, we defined a novel parameter, the intrinsic calcification angle (ICA), as the arc externally (i.e. abluminally) projected by a calcification; a sample measurement is demonstrated in Fig. 1. This has to be kept distinct from the calcium arc, which measures the dimension of every calcification as the arc of vessel circumference (Additional file 2: Figure S2). ICA, on the contrary, is a parameter assessing calcification geometry. In case of circumferential or irregular calcifications, the smallest ICA for every OCT-section was considered; examples of the measurement of such ICAs are shown in Additional file 2: Figure S2. Intra- and interobserver variability for minimal ICA were 0.965 and 0.957, respectively.

### Finite elements structural analysis

In order to gain mechanistic insight and to analyze the effects of changes in ICA on the mechanics of the coronary plaque, we performed finite elements structural analysis using commercially available software. We simulated the vessel as a hollow cylinder with a lumen diameter of 1 mm and a vessel diameter of 3 mm. In analogy to previous models [7, 19], we simulated calcifications as rigid inclusions in an elastic material. We considered calcifications as compressible materials (with a Poisson`s ratio of 0.3) and the vessel wall as a nearly uncompressible material (with a Poisson’s ratio of 0.45). The Young’s modulus of the calcification (10 GPa) and of the vessel wall (500 kPa) as well as the density of the calcification (2700 kg/m^3^) and of the vessel wall (1050 kg/m^3^) were extrapolated from previous literature [7, 19]. In order to exclude an influence of other parameters of calcification morphology on the peak cap stress, we initially set a calcium arc of 60°, a depth of calcification of 100 µm and a calcification thickness of 0.5 mm as constants in the performed simulations. In order to standardize the calcium arc and to be able to variate the ICA without affecting other parameters of calcification morphology, we proposed a structure composed of an ellipsoid with a more superficial tetrahedron embedded in it. A pressure of 110 mmHg (= 14.6 kPa) on the luminal side was applied as external load onto the lumen surface and a simulation of the structural stress in response to the load was performed. We then analyzed the stress distribution as von Mises stress in a bi-dimensional cross section of the vessel normal to the vessel axis, which included the calcification. A graphical representation of the model is included in the Additional file 3: Figure S3. The stress intensity on the fibrous cap was graphically shown on a blue-red color scale and plotted against the distance from the lumen. The highest von Mises stress in the considered section was considered as peak cap stress.

Furthermore, in order to better characterize the impact of the various morphologic features of calcification on the stress increase due to ICA, we performed two similar analyses assuming ICAs of 20°, 40° and 70° as constant and variating respectively the depth of the calcification (in the range 50–500 µm) and the calcium arc (in the range 60°−80°). As outcomes we measured the absolute stress intensity and (only for the simulation assessing the depth of calcification) the stress concentration at 5 µm distance from the lumen.

### Statistical analysis

Categorical variables were summarized as count (percentage), continuous variables as mean ± standard deviation. Distributions of continuous variables were compared with *t* test. The association of categorical variables was evaluated by Pearson’s Chi squared test. In statistical testing, we did not account for multiple calcifications in the same segment. To identify the optimal cut-off-value of minimal and mean ICA to predict an ACS, we performed a receiver operating curve (ROC) analysis. Values with the highest Youden-index were identified as optimal cut-off-values. A classification of the diagnostic efficiency according to the values of the area under the curve (AUC) was used as described elsewhere [22]. Univariate logistic regression analysis was performed to investigate which parameters predict a low ICA. Test results are reported as p-value (p), odds ratio (OR) and corresponding 95% confidence intervals (CI). Uni- and multivariate logistic regression analysis was also performed to investigate if mean ICA and mean ICA < 175.9° predicts ACS independently from classical parameters of plaque vulnerability. Therefore, mean ICA was adjusted for minimal FCT, lipid volume index and the presence of macrophages, which are known to be determinants for the presence of ACS [23]. Mean ICA < 175.9° was not included in the multivariable model due to collinearity with mean ICA. All statistical analyses were performed with SPSS software (IBM Corp., Armonk, NY, USA). Statistical significance was awarded by p < 0.05.

## Results

### Population characteristics

Patients with ACS and stable CAD did not significantly differ with respect to classical cardiovascular risk factors, complications of diabetes or medication. However, a higher HbA1c (7.7 ± 1.8% vs. 7.2 ± 1.6%, p = 0.008) and a trend towards a higher CRP (20.1 ± 31.6 vs. 9.3 ± 11.6 mg/dl, p = 0.057) could be detected in patients with ACS compared to patients with stable CAD. Patients with ACS showed a thinner minimal (51.3 ± 9.4 vs. 80.4 ± 27.0 µm, p < 0.001) and mean (97.2 ± 18.0 vs. 125.7 ± 29.5 µm, p < 0.001) fibrous cap thickness, a larger lipid volume index (1105.4 ± 343.8 vs. 493.8 ± 361.2 mm*°, p < 0.001) and more often plaques with macrophages (25 (69.4%) vs. 23 (41.1%), p = 0.002) compared to patients with stable CAD. Further details are reported in Table 1.

**Table 1:** Population analysis

	Stable CAD (n = 56)	ACS (n = 36)	P
Age (years)	70.4 ± 5.9	67.8 ± 9.2	0.171
Sex (male, n, %)	36 (64.3)	25 (69.4)	0.609
Hypertension (n, %)	49 (87.5)	23 (63.9)	0.365
Systolic BP (mmHg)	137.8 ± 19.6	137.0 ± 16.6	0.827
Current nicotine use (n, %)	9 (16.1)	10 (27.8)	0.176
Total pack years (pack years)	17.1 ± 22.2	18.2 ± 17.3	0.796
Positive family history (n, %)	25 (44.6)	12 (33.3)	0.280
Hyperlipidaemia (n, %)	39 (69.6)	23 (63.9)	0.566
BMI (kg/m^2^)	30.8 ± 4.6	29.4 ± 6.0	0.218
COPD (n, %)	6 (10.7)	5 (13.9)	0.647
Known CAD (n, %)	20 (35.7)	13 (36.1)	0.969
Previous PCI (n, %)	14 (25.0)	13 (36.1)	0.559
Previous CABG (n, %)	2 (3.6)	1 (2.8)	0.834
Diabetes severity			
Duration of diabetes (years)	11.4 ± 9.5	11.3 ± 10.5	0.943
History of diabetic retinopathy (n, %)	10 (17.9)	8 (22.2)	0.606
History of diabetic neuropathy (n, %)	20 (35.7)	8 (22.2)	0.170
HbA1c (%)	7.2 ± 1.6	7.7 ± 1.8	0.008
Lab values			
Total cholesterol (mg/ml)	191.2 ± 45.1	187.1 ± 44.3	0.667
LDL-cholesterol (mg/ml)	117.8 ± 36.4	113.0 ± 36.2	0.543
HDL-cholesterol (mg/ml)	44.2 ± 10.3	42.5 ± 12.4	0.472
Triglycerides (mg/dl)	181.8 ± 103.2	165.4 ± 95.2	0.454
eGFR (ml/min/1.73 m^2^)	58.8 ± 6.5	58.1 ± 6.4	0.620
BUN (mg/dl)	40.6 ± 14.5	40.2 ± 16.5	0.899
Serum Creatinine (mg/dl)	1.0 ± 0.2	1.0 ± 0.3	0.927
CRP (mg/ml)	9.3 ± 11.6	20.1 ± 31.6	0.057
Creatine kinase at admission (U/l)	102.9 ± 60.1	190.1 ± 158.3	0.003
LDH (U/l)	213.2 ± 66.4	245.4 ± 82.0	0.046
Maximal troponin T (ng/ml)	NA	487.4 ± 579.5	NA
Maximal CK (U/l)	NA	283.1 ± 215.7	NA
Maximal CK-MB (U/l)	NA	84.0 ± 16.9	NA
Medication pre-OCT			
Aspirin (n, %)	52 (92.9)	32 (88.9)	0.510
Betablocker (n, %)	46 (82.1)	25 (71.4)	0.230
ACE-i or ARB (n, %)	39 (72.2)	24 (68.8)	0.711
Insulin (n, %)	26 (46.6)	14 (38.9)	0.476
Metformin (n, %)	37 (66.1)	22 (61.1)	0.628
Sulfonylureas (n, %)	13 (23.2)	5 (13.9)	0.271
Incretins (n, %)	12 (21.4)	6 (16.7)	0.574
Statin (n, %)	40 (71.4)	22 (61.1)	0.354
Plaque features in OCT			
Minimal FCT (µm)	80.4 ± 27.0	51.3 ± 9.4	< 0.001
Mean FCT (µm)	125.7 ± 29.5	97.2 ± 18.0	< 0.001
Lipid volume index (mm*°)	493.8 ± 361.1	1105.4 ± 343.8	< 0.001
Presence of macrophages (n, %)	23 (41.1)	25 (69.4)	0.002

*ACEi* ACE-inhibitors, *ACS* acute coronary syndrome, *BMI* body mass index, *BP* blood pressure, *ARB* angiotensin receptor blockers, *CABG* coronary artery bypass graft, *CAD* coronary artery disease, *CK* creatin-kinase, *CK-MB* creatin-kinase muscle-brain, *CRP* C-reactive protein, *FCT* fibrous cap thickness, *eGFR* estimated glomerular filtration rate, *HDL* high density lipoprotein, *LDH* lactate dehydrogenase, *LDL* low density lipoprotein, *PCI* percutaneous coronary intervention

### ICA and further CAC morphology

**Fig. 2:**
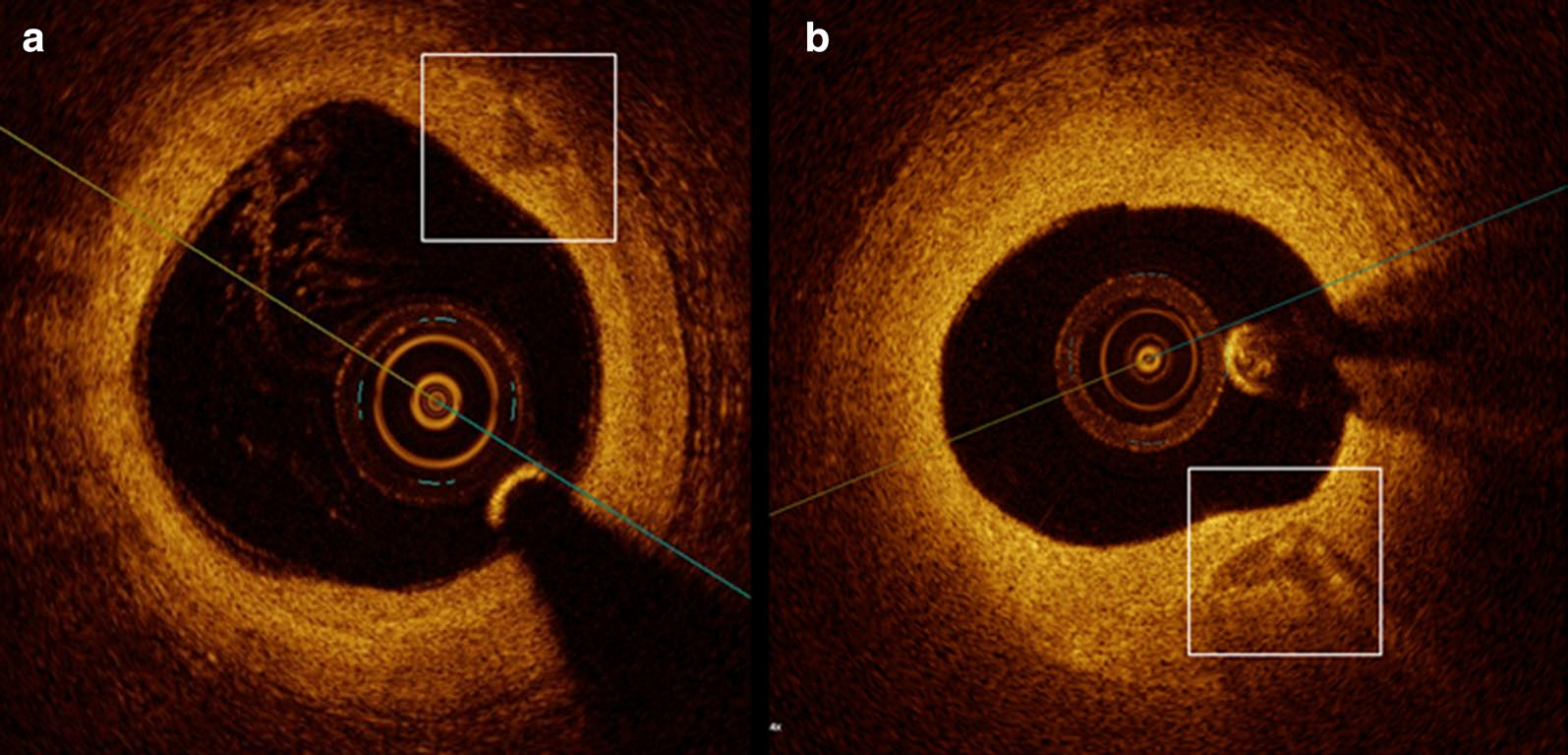
Representative calcification morphology in patients with ACS and stable CAD. A lower ICA is seen in a calcification of a patient with ACS (**a**) in comparison to a calcification of a patient with stable CAD (**b**). *ICA* intrinsic calcification angle, *ACS* acute coronary syndrome, *CAD* coronary artery disease

Calcifications in patients with ACS demonstrated a significantly lower minimal (139.8 ± 32.8° vs. 165.6 ± 21.6°, p < 0.001) and mean ICA (164.1 ± 14.3° vs. 176.0 ± 8.4°, p < 0.001) as compared to patients with stable CAD. Representative images are shown in Fig. 2. Other parameters of CAC were not different between patients with ACS compared to patients with stable CAD (Table 2). We found no difference in lesion localization in minimal (LAD 160.4 ± 25.2°; LCX 155.1 ± 29.3°; RCA 148.5 ± 33.2°) or mean ICA (LAD 173.5 ± 11.1°; LCX 170.2 ± 12.8°; RCA 168.8 ± 14.0° all p = NS).

**Table 2:** Analysis of calcification morphology

	Stable CAD (n = 219)	ACS (n = 143)	p
Spotty calcification (n, %)	131 (59.8)	85 (59.4)	0.925
Mean calcium arc (°)	61.3 ± 40.5	62.8 ± 35.3	0.721
Maximal calcium arc (°)	92.7 ± 76.0	91.1 ± 65.1	0.836
Mean calcium depth (µm)	152.4 ± 99.0	146.0 ± 105.9	0.560
Minimal calcium depth (µm)	99.9 ± 97.1	96.6 ± 98.2	0.759
Mean calcium area (mm^2^)	0.64 ± 0.62	0.67 ± 0.52	0.610
Maximal calcium area (mm^2^)	1.17 ± 1.38	1.12 ± 1.03	0.681
Calcium length (mm)	1.77 ± 2.30	1.45 ± 1.42	0.101
Calcium volume index (°*mm)	170.0 ± 392.8	113.7 ± 172.9	0.063
Mean ICA (°)	176.0 ± 8.4	164.1 ± 14.3	< 0.001
Minimal ICA (°)	165.6 ± 21.6	139.8 ± 32.8	< 0.001

*ICA* intrinsic calcification angle

To evaluate the diagnostic value of ICA to predict ACS, logistic regression analysis was performed. Both minimal (OR 0.76 per 10° increase, 95% CI 0.69–0.83, p < 0.001) and mean ICA (OR 0.30 for 10° increase, 95% CI 0.22–0.41, p < 0.001) predicted the presence of ACS.

**Fig. 3:**
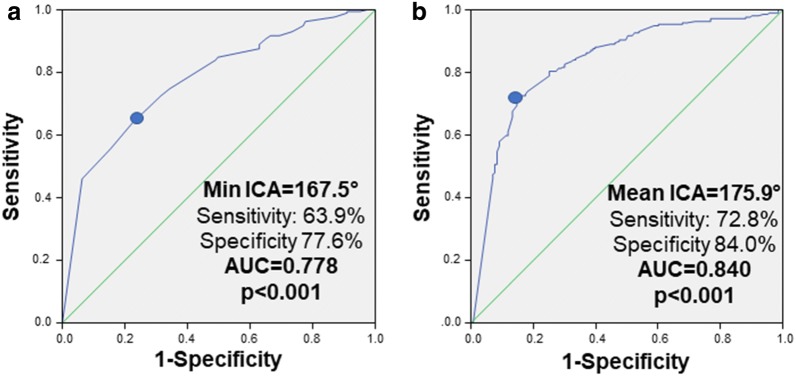
Intrinsic calcification angle (ICA) as determinant of ACS. Receiver operating curves for the prediction of ACS for minimal (**a**) and mean (**b**) ICA. Abbreviation as in Fig. 2

Next, ROC-analyses were performed to evaluate the diagnostic efficiency of ICA to differentiate ACS from stable CAD. In the ROC-analysis reported in Fig. 3, we found that both minimal (AUC 0.778, 95% CI 0.731–0.826, p < 0.001) and mean ICA (AUC 0.840, 95% CI 0.797–0.882, p < 0.001) differentiated ACS from stable CAD with good/very good diagnostic efficiency. The optimal cut-off-values to individuate ACS were 167.5° (sensitivity at optimal cut-off: 63.9%, specificity at optimal cut-off 77.6%) for minimal and 175.9° (sensitivity at optimal cut-off: 72.8%, specificity at optimal cut-off 84.0%) for mean ICA. In univariate logistic regression analysis, a younger age (OR 0.95 per year, 95% CI 0.92–0.98, p = 0.002), male sex (OR 2.18, 95% CI 1.41–3.38, p < 0.001), lower HDL-cholesterol (OR 0.82 per 10 mg/dl, 95% CI 0.68–0.98, p = 0.029) and ACS (OR 14.71, 95% CI 8.47–25.64, p < 0.001) significantly predicted the presence of a mean ICA < 175.9°. Further data are reported in Table 3.

**Table 3:** Univariate logistic analysis of the determinants of a mean ICA lower than 175.9°

	OR (95% CI)	p
Clinical parameters		
Age (per year)	0.95 (0.92–0.98)	0.002
Sex (male)	2.18 (1.41–3.38)	< 0.001
ACS at presentation (present)	14.71 (8.47–25.64)	< 0.001
Hypertension (present)	0.75 (0.39–1.47)	0.408
Systolic BP (per 10 mmHg)	0.98 (0.88–1.11)	0.797
Current nicotine use (present)	1.11 (0.65–1.89)	0.703
Total pack years (per 10 pack years)	1.06 (0.93–1.20)	0.374
Positive family history (present)	0.68 (0.45–1.04)	0.078
BMI (per kg/m^2^)	1.00 (0.97–1.03)	0.998
COPD (present)	0.59 (0.30–1.17)	0.135
Known CAD (present)	0.88 (0.57–1.35)	0.556
Previous PCI (present)	1.20 (0.76–1.91)	0.430
Previous CABG (present)	0.43 (0.15–1.28)	0.132
Diabetes severity		
Duration of diabetes (present)	1.00 (0.98–1.02)	0.840
History of diabetic retinopathy (present)	1.40 (0.81–2.40)	0.228
History of diabetic neuropathy (present)	1.02 (0.65–1.60)	0.936
HbA1c (per %)	0.97 (0.85–1.10)	0.634
Lab values		
Total cholesterol (per 10 mg/ml)	0.98 (0.93–1.03)	0.406
LDL-cholesterol (per 10 mg/ml)	0.97 (0.91–1.03)	0.379
HDL-cholesterol (per 10 mg/ml)	0.82 (0.68–0.98)	0.029
Triglycerides (per 10 mg/dl)	0.99 (0.97–1.01)	0.277
eGFR (per 10 ml/min/1.73 m2)	1.05 (0.75–1.46)	0.772
CRP (per 10 mg/ml)	1.07 (0.95–1.21)	0.259

To investigate if ICA is associated with ACS independently from classical features of the vulnerable plaque such as fibrous cap thickness, presence of macrophages and necrotic lipid core, multivariate logistic regression analysis was performed. In this analysis mean ICA was still and independently associated with ACS (OR for 10°-variation 0.25 (95%-CI 0.13–0.52), p < 0.001). See Table 4 for further details.

**Table 4:** Morphologic determinants of ACS in uni- and multivariate analysis

	OR	p
Univariate analysis		
Mean ICA (per 10°)	0.30 (0.22–0.41)	< 0.001
Mean ICA < 175.9°	14.7 (8.50–25.5)	< 0.001
Minimal FCT (per 10 µm)	0.34 (0.24–0.48)	< 0.001
Presence of macrophages (present)	1.68 (1.09–2.61)	0.020
Lipid volume index (per 100 mm*°)	1.46 (1.32–1.61)	< 0.001
Multivariate analysis		
Mean ICA (per 10°)	0.25 (0.13–0.52)	< 0.001
Minimal FCT (per 10 µm)	0.38 (0.25–0.59)	0.001
Presence of macrophages (present)	3.09 (1.19–8.05)	0.021
Lipid volume index (per 100 mm*°)	1.24 (1.10–1.40)	0.001

### Plaque rupture or calcified nodules and distance to minimal intrinsic angle

Of the 36 analyzed segments in patients with an ACS, we could detect a plaque rupture in 26 (72.2%) and a calcified nodule in 4 (11.1%). 24 calcifications were co-localized with plaque ruptures (14.2% of the calcifications in ACS patients), with 12 presenting as plaque ruptures alone (7.1%) and 12 presenting as calcified nodules (7.1%). The mean longitudinal distance between the sites of plaque rupture/calcified nodule and the minimal intrinsic angle in the considered calcification was 0.4 ± 0.5 mm (with an average length of calcification of 2.3 ± 2.9 mm). The site of the plaque rupture or calcified nodule was in 5 cases (20.8%) coincident with the site presenting the minimal ICA, and in further 9 cases (37.5%) only 0.2 mm away from it.

### Finite elements structural analysis

**Fig. 4:**
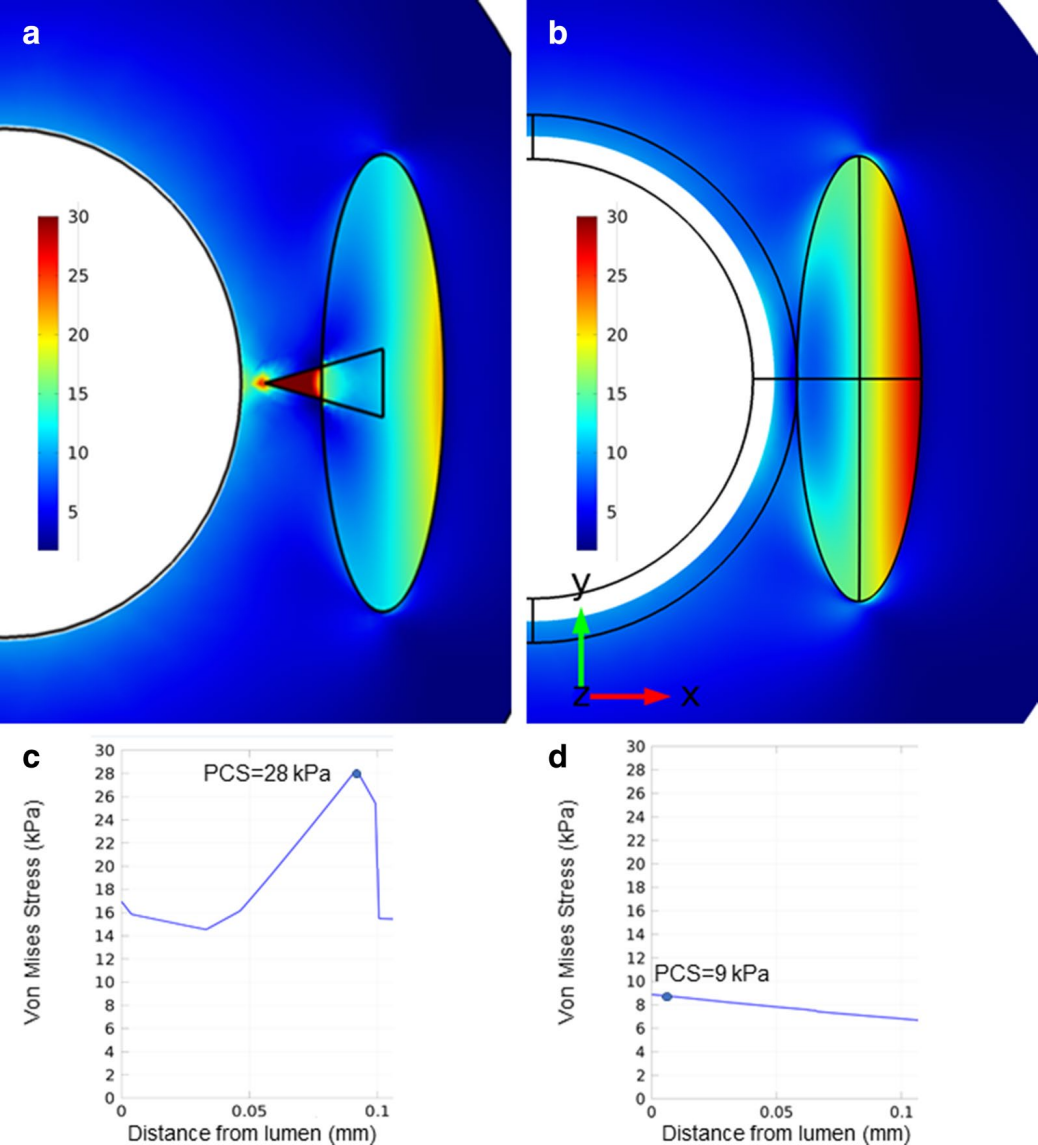
Stress on the fibrous cap in dependence of the ICA in a finite elements structural analysis. A higher PCS can be seen in the fibrous cap directly overlying a calcification with a smaller ICA (30°, **a**) as compared to a calcification with an ICA of 180° (**b**). Spatial stress distributions are reported in C and D for these two scenarios. *ICA* intrinsic calcification angle, *PCS* peak cap stress. In **a** and **b**, vessel lumen is shown in white, and a calcification with different morphology is embedded in the vessel wall and contoured with a black line

After we found that a low ICA of a coronary artery calcification is associated with ACS, finite elements structural analysis was performed to gain mechanistic insight into how changes in ICA as well as changes in depth of calcification affect differences in fibrous cap stress. First, a small (30°) and large (180°) ICA were compared. In this analysis, we measured an up to 310% higher stress concentration (28 kPa for ICA = 30° vs. 9 kPa for ICA = 180°) in the presence of a smaller ICA as shown in Fig. 4a, b. The peak stress on the fibrous cap was exerted in the portion of the vessel wall immediately overlying the calcification. The spatial distributions are simulated and depicted in Figs. 4c, d.

**Fig. 5:**
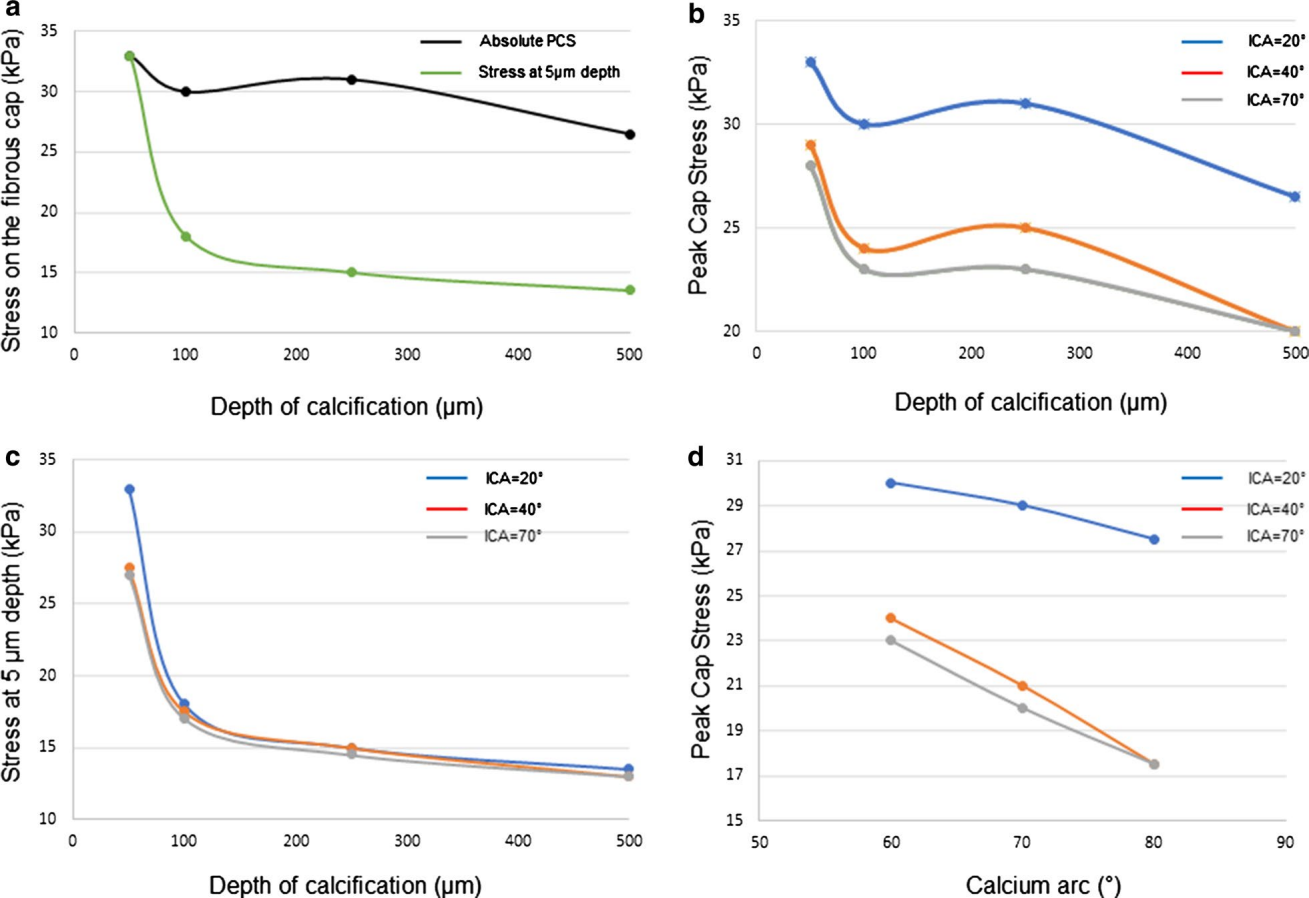
Variation of the ICA-induced stress in dependence of calcification depth and dimensions. In (**a**) more superficial calcifications cause a higher absolute peak cap stress (black line) and, more importantly, a higher stress concentration at 5 µm depth from the lumen (green line). These effects remain consistent analyzing PCS (**b**) and stress at 5 µm depth (**c**) in a range of ICAs varying from 20° to 70°. The stress exerted on the fibrous cap by a calcification with a small ICA is also inversely related to the dimensions of the calcification itself, which is consistent throughout the whole range of analyzed ICAs (**d**). Abbreviation as in Fig. 4

Next, we investigated the impact of calcification depth on peak cap stress. In these analyses, more superficial calcifications caused higher absolute peak cap stress, as shown in Fig. 5a, b. As fibrous cap stress close to the luminal surface of the vessel wall is relevant for fibrous cap/plaque rupture, fibrous cap stress was investigated at 5 µm depth. This analysis reveals that the stress on the fibrous cap at 5 µm is potentiated if depth of calcification is reduced (33 kPa for depth of 50 µm vs. 18 kPa for depth of 100 µm, 15 kPa for depth of 250 µm and 13 kPa for depth of 500 µm). This relationship is depicted in Fig. 5a for an ICA of 20° and remained consistent also in simulations with different ICAs, as shown in Figs. 5b, c.

Finally, we tested if the relationship between ICA and fibrous cap stress is dependent on the size of calcification. As demonstrated in Fig. 5d, a smaller calcium arc increases the peak cap stress. However, the peak cap stress increases with smaller ICAs in all calcium arcs investigated, suggesting that the effect of ICA on the peak cap stress adds to the increased stress exerted by smaller calcifications.

## Discussion

In this OCT analysis in patients with T2DM, we could demonstrate that coronary calcifications of patients with ACS present a significantly lower ICA compared to calcifications in patients with stable CAD. Lower ICA is a determinant of ACS independently from other features of plaque vulnerability. Furthermore, finite elements structural analysis demonstrated that this novel morphological parameter increases the stress on the fibrous cap. This phenomenon is potentiated if calcifications are smaller and more superficial.

So far, analyses regarding the impact of CAC on plaque vulnerability focused on the dimensions of calcifications, suggesting that smaller calcifications contribute to plaque vulnerability [4–8, 14–18] in addition to other established parameters [23]. In the present study, we extend the current knowledge by demonstrating that a lower ICA is more frequently found in the culprit segments of patients with ACS than in the target segments of patients with stable CAD, suggesting that ICA may be a novel feature of the vulnerable plaque. We and others previously demonstrated that vulnerable plaques are characterized by a lower minimal FCT, a higher lipid volume index and a higher frequency of macrophage infiltration compared to stable plaques [23, 24]. With regard to minimal FCT, interventional studies demonstrated an increase of minimal FCT using statin treatment [25, 26], suggesting that at least some of these features of plaque vulnerability may be influenced by pharmacological interventions. Further studies are necessary to evaluate, if ICA responds to pharmacological treatment and reduces plaque vulnerability in patients.

We selected a cohort of patients with T2DM to assess the relevance of ICA as a novel feature of plaque vulnerability, provided that these patients more frequently exhibit a high-risk plaque phenotype [12, 24, 27, 28], which even starts with pre-diabetic conditions [29]. Often, patients with T2DM first present with more advanced CAD at ACS [30]. However, the existence of a relationship between T2DM and CAC morphology is still incompletely understood: some studies highlighted a more extensive calcification in the presence of T2DM [11, 28], while others showed no relevant difference in CAC morphology in patients with and without T2DM [12].

### Finite elements analysis

Our observations were confirmed in finite elements structural analysis, in which we found a striking increase of the peak cap stress on the fibrous cap in the presence of a smaller ICA. It should be noted that the absolute increase is modest and that the calculated peak cap stress still remains below the critical threshold for rupture, which has been estimated at 300 kPa [31]. However, our finite elements model only takes into account the calcification and its intrinsic angle as a potential destabilizing factor. Thus, in the context of a typical vulnerable plaque with a thin fibrous cap and a large necrotic core, the threefold increase in peak cap stress arising from a lower ICA could be more than sufficient to exceed the threshold for rupture at 300 kPa.

Furthermore, we characterized how the relationship between ICA and peak cap stress is influenced by other morphological features of calcification. We could demonstrate that more superficial calcifications cause a higher stress concentration in terms of absolute values and—even more relevant—a higher stress concentration in the most superficial layers of the fibrous cap. This superficial stress concentration may therefore predispose to rupture of the fibrous cap and subsequently cause an ACS. Interestingly, an almost twofold increase of the stress exerted at 5 µm occurs for calcification with a depth of 50 µm compared to calcifications with a depth of 100 µm, suggesting that slight variations in this range may play a significant biological role.

We also found an inverse correlation between the dimensions of the calcification and the peak cap stress, confirming the destabilizing effect of smaller calcifications which is in line with the findings of previous studies [5–9, 14–18]. Therefore, we can conclude that a smaller ICA of calcified particles under certain circumstances (i.e., a more superficial and/or less extensive calcification) poses an even greater risk for rupture by further increasing fibrous cap stress.

### Potential clinical relevance of ICA

Next, we analyzed the diagnostic efficiency of mean and minimal ICA to predict ACS. We found mean ICA to be a better determinant for ACS compared to minimal ICA. The clinical parameters predicting a mean ICA below the proposed best cut-off-value (175.9°) give further insight into this new morphological feature of plaque vulnerability. In fact, some classical risk factors for atherosclerosis including male sex and a low HDL-cholesterol predicted the presence of lower ICA. Taken together, this novel feature of plaque vulnerability may be present more frequently in a subpopulation of patients with increased cardiovascular risk. Interestingly, a lower ICA is also predicted by a younger age, a finding which may prompt further research on the evolution of CAC over time and on its relationship with plaque vulnerability. Furthermore, the role of CAC morphology rather than CAC quantification alone may explain some paradoxes such as the higher CAC scores presented by endurance athletes compared to age-adjusted inactive subjects on coronary CT [32]. In this light, vulnerability may not only be determined by the extent of calcification, but rather by its distribution and geometry—a hypothesis not only linked to ICA, but for instance also to the size of the calcifications (i.e., spotty/micro-calcifications). On the other hand, the apparent paradox of a more frequent subclinical CAD in endurance athletes may also be explained by possible detrimental effects of excess physical activity.

In the light of both our mechanistic and clinical data, it is tempting to speculate that ICA relevantly contributes to the development of ACS. As such, arrow-shaped vascular calcifications with a low ICA may pose a threat to fibrous caps with subsequent plaque rupture resulting in ACS. However, ICA is only one of various morphologic features of plaque vulnerability and the interplay of all these factors should be taken into account when evaluating the net stability/vulnerability of a lesion.

### Limitations

As this is the first study to define ICA and to evaluate its impact on the vulnerability of coronary plaques, our results have to be confirmed in larger populations, generalized to other study populations (e.g. non-diabetic patients) and evaluated in prospective trials. Particularly the limited patient number may affect the outcomes of our clinical study part with regard to some morphological aspects of CAC such as its extension or the depth of the calcifications. These parameters may have a relevant role in the genesis of plaque vulnerability as our simulation data suggest. Although our finite elements structural analysis data provide additional valuable mechanistic insight into how ICA impacts on plaque vulnerability, the observational nature of the clinical study part cannot prove causality. Moreover, as patients with kidney disease, who are in particular prone to vessel calcification, were excluded due to ethical reasons as the OCT investigation requires additional contrast media, we cannot draw any conclusion regarding this subpopulation. Furthermore, as patients with severely calcified and tortuous lesions were not included in the study as this often does not allow the safe advancement of the OCT-catheter, we cannot exclude a potential selection bias with regard to the clinical part of this study. Besides, we cannot exclude any change in CAC morphology due to lesion pre-dilatation. However, pre-dilatation was only performed if necessary for the safe advancement of the OCT catheter in rare cases and profound changes in ICA are unlikely due to mechanical properties of CAC.

Furthermore, the finite elements model employed required several simplifying assumptions and was only elaborated to analyze the influence of ICA on the vessel mechanics. Moreover, although the data showing a higher stress caused by low ICA in smaller calcifications are in line with the present literature, our analysis could be performed in a relatively small range of calcium arcs due to geometric limitations of the proposed model.

## Conclusions

Our clinical and mechanistic data for the first time suggest ICA to be a novel feature of coronary plaque vulnerability. The impact of ICA on fibrous cap stress is potentiated in more superficial calcifications and adds to the destabilizing role of smaller calcifications.

## Supplementary information

**Additional file 1: Fig. S1.** Details of screening and inclusion process.

**Additional file 2: Fig. S2.** Assessment of ICA in case of complex calcium morphology. For every OCT-section, the smallest ICA was recorded. For each calcification, we also show calcium arc (in yellow).

**Additional file 3: Fig. S3.** Methods of the finite elements structural analysis. Calcification was simulated as a solid inclusion in the context of a cylindrical vessel wall (A). The considered dimensions are reported in (B). The mesh was then generated, as shown in (C), and stresses were analyzed on a *xy* plane intersecting the inclusion (D).

## Data Availability

Data are available on request to the corresponding author.

## Abbreviations

ACS: acute coronary syndrome
CAC: coronary artery calcification
CAD: coronary artery disease
ICA: intrinsic calcification angle
IVUS: intravascular ultrasound
OCT: optical coherence tomography
T2DM: type 2 diabetes mellitus
