# Supplementary figures and images for: Intrinsic calcification angle: a novel feature of the vulnerable coronary plaque in patients with type 2 diabetes: an optical coherence tomography study
Source: Cardiovasc Diabetol. 2019 Sep 24;18:122. doi: 10.1186/s12933-019-0926-x (PMC6760065; doi:10.1186/s12933-019-0926-x)

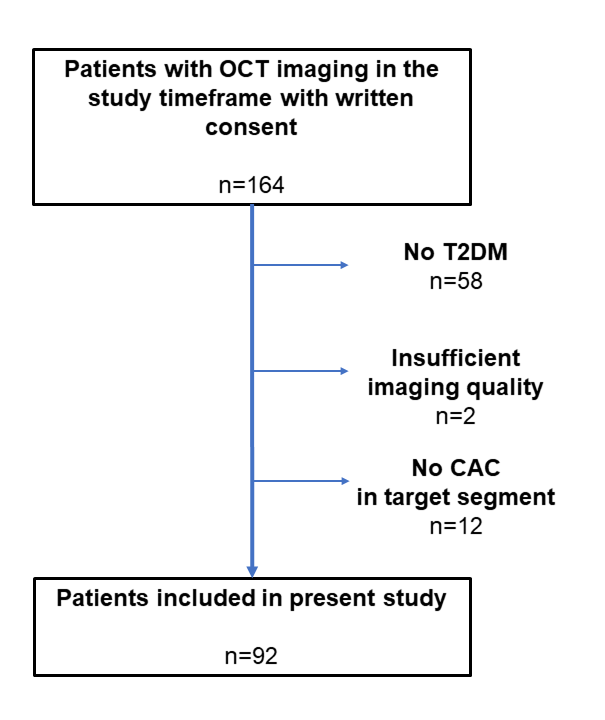

Supplement: Supplementary file 1 — Additional file 1: Fig. S1. Details of screening and inclusion process. [file 12933_2019_926_MOESM1_ESM.tif]

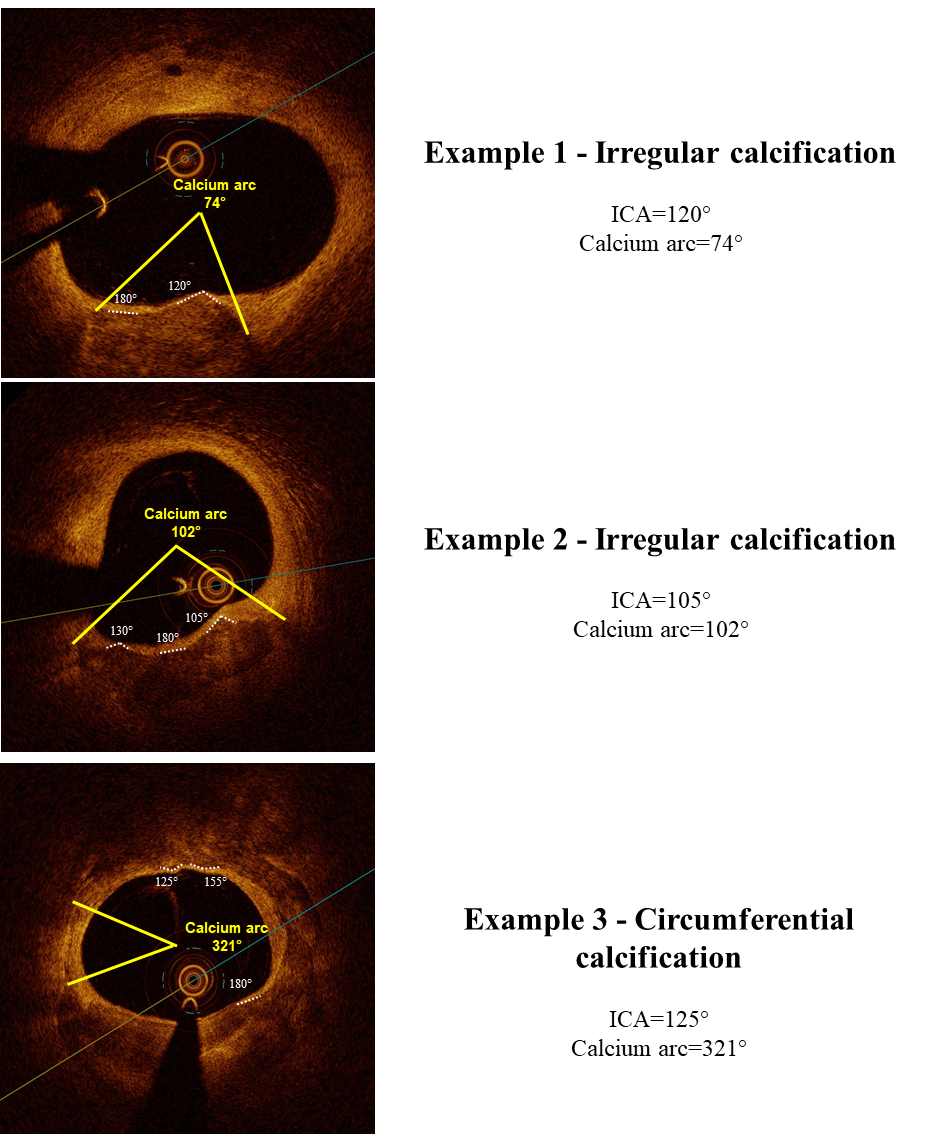

Supplement: Supplementary file 2 — Additional file 2: Fig. S2. Assessment of ICA in case of complex calcium morphology. For every OCT-section, the smallest ICA was recorded. For each calcification, we also show calcium arc (in yellow). [file 12933_2019_926_MOESM2_ESM.tif]

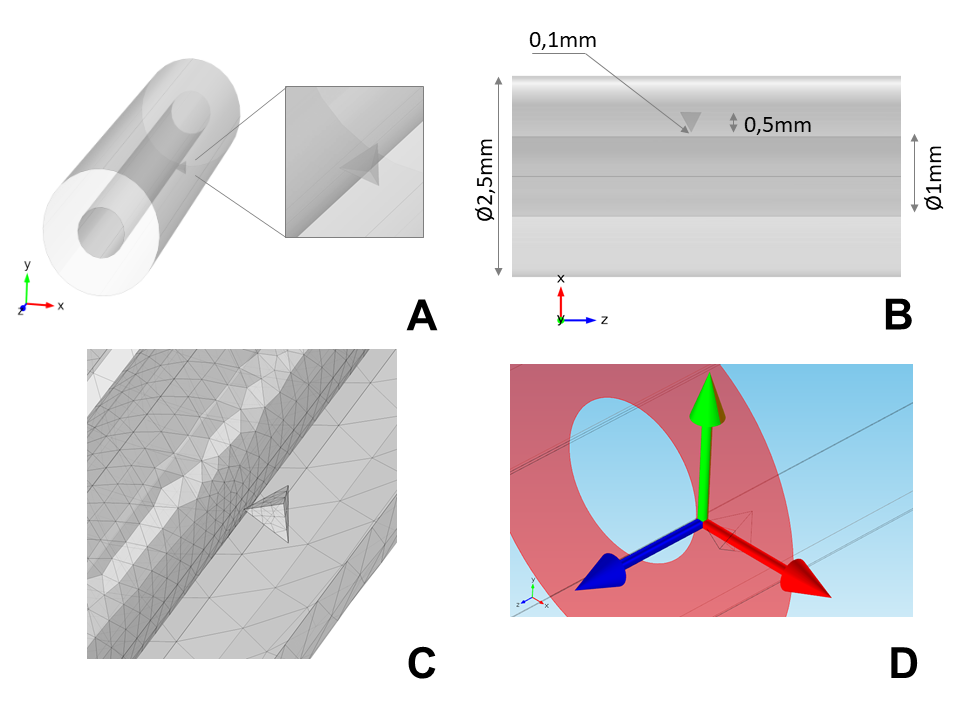

Supplement: Supplementary file 3 — Additional file 3: Fig. S3. Methods of the finite elements structural analysis. Calcification was simulated as a solid inclusion in the context of a cylindrical vessel wall (A). The considered dimensions are reported in (B). The mesh was then generated, as shown in (C), and stresses were analyzed on a xy plane intersecting the inclusion (D). [file 12933_2019_926_MOESM3_ESM.tif]
